# Study of long-term effects of pelvic radiotherapy on the function of bone marrow in recurrent cervical cancer patients

**DOI:** 10.7150/ijms.95900

**Published:** 2024-08-01

**Authors:** Boer Deng, Weimin Kong, Chao Han, Chunxiao Zhou, Jing Li, Dan Song, Yuxuan Lin

**Affiliations:** 1 Department of Gynecology, Beijing Obstetrics and Gynecology Hospital, Capital Medical University. Beijing Maternal and Child Health Care Hospital, Beijing 100006, P.R. China.; 2 Division of Gynecologic Oncology, University of North Carolina at Chapel Hill, Chapel Hill, NC, USA.; 3 Lineberger Comprehensive Cancer Center, University of North Carolina at Chapel Hill, Chapel Hill, NC, USA.

**Keywords:** Cervical cancer, radiotherapy, relapse, bone marrow suppression

## Abstract

**Purpose:** To study the effects of prior pelvic radiotherapy on bone marrow suppression in recurrent cervical cancer patients during chemotherapy.

**Methods and materials:** The cases of 129 patients with recurrent cervical cancer were reviewed, of which 77 patients had pelvic radiotherapy history and another 52 patients with no pelvic radiotherapy history were used as control group. All patients received a chemotherapy regimen of paclitaxel combined with carboplatin (TC) per 21 days for 5-6 times. Hematologic toxicity, including count of red blood cell, white blood cell and neutrophil cell and platelet, was defined by using Common Terminology Criteria for Adverse Events (version 4.0). The relationship between age, body mass index, disease free survival, pathological types, FIGO stages, radiotherapy methods and the degree of bone marrow suppression during chemotherapy was statistically analyzed, respectively, for all recurrent cervical cancer patients. **Results:** Among 77 patients with previous radiotherapy history, 73 recurrent patients (94.8%) had bone marrow suppression followed by chemotherapy. Recurrent cervical cancer patients without prior radiotherapy (n=52) showed a lower risk of bone marrow suppression followed by chemotherapy (n=39, 75.0%, P < 0.05). The probability of severe bone marrow suppression (grade III-IV) after chemotherapy in recurrent cervical patients with or without history of radiotherapy was 41.6% and 13.5%, respectively (P < 0.05). In univariate analysis, radiotherapy methods were associated with the incidence of grade III-IV bone marrow suppression in recurrent cervical cancer patients (P=0.005). In multivariate analysis, radiotherapy methods and extended-field radiotherapy were the risk factor of grade III-IV bone marrow suppression (χ^2^=16.975, P=0.001). No significant differences in the counts of white blood cell, hemoglobin and platelet were observed before chemotherapy at relapse between patients with and without prior radiotherapy. Reduction of white blood cell counts, absolute value of neutrophil cell and platelet counts composited majority type of grade III and IV bone marrow suppression.

**Conclusions:** The prior pelvic radiotherapy significantly increased the incidence of bone marrow suppression during chemotherapy in recurrent cervical cancer patients. When treating recurrent cervical cancer patients with chemotherapy who had prior radiotherapy, especially for those experienced external beam radiation therapy, essential attention and timely intervention are recommended to ensure completion of chemotherapy and clinical efficacy.

## Introduction

Cervical cancer is the fourth most common cancer in incidence and mortality in women worldwide with about 13,960 new cases diagnosed and 4,310 cases dead from cervical cancer in United state in 2023 1. Several risk factors, such as human sustainable papillomavirus (HPV) infection, smoking, active sexual history, and long-term use of oral contraceptives, increase the chance of developing cervical cancer 2. With the cancer progression, cervical cancer commonly causes local spread (pelvic, vaginal), lymph node metastasis and other distant metastasis (lung, bone, brain) 3, 4. Surgery and radiotherapy are the main two therapeutic strategies for treating cervical cancer and chemotherapy is employed as alternative adjuvant therapy according to the risk factors individually. For early stages of cervical cancer, either surgery or radiation combined with chemotherapy lead to a well prognosis with 60-70% five-year survival. For later stages, radiotherapy combined with chemotherapy is the most recommended treatment strategy with a relatively poor prognosis compared to patients with early stages 5. The patients with cervical cancer recurrence usually exhibit poor prognosis and high mortality rates and nearly 30% patients with invasive carcinoma die owing to recurrence or metastasis 6, 7. Notably, due to the lack of effective treatment strategies, the treatment of recurrent cervical cancer patients still faces significant clinical challenges. In addition to surgery, radiotherapy and chemotherapy are usually used in the treatment of recurrent cervical cancer patients, attention needs to be paid to whether these patients are more prone to bone marrow suppression 5, 8.

Radiotherapy is the most-effective cytotoxic therapy in treating patients with cervical cancer 9. Concurrent chemoradiotherapy is the standard strategy for cervical cancer patients with stage IB3 to IVA disease 10. Radiotherapy for patients with cervical cancer includes external beam radiation therapy (EBRT) and brachytherapy, and the dose of radiation for EBRT and brachytherapy for patients with cervical cancer are commonly 40-45 Gary (Gy) and 30-40 Gy, respectively, according to the National Comprehensive Cancer Network (NCCN) guidelines. EBRT is considered to have certain hematologic toxicity (HT) for cancer patients since the approximately 50% of active bone marrow is located in pelvic and lower spine bones and active bone marrow is the most sensitive tissue to ionizing radiation, thereby increasing the risk of bone marrow suppression especially when combined with chemotherapy with inhibitory effects on bone marrow function 11-14. Furthermore, the range of irradiation field and dose of irradiation may need to be expanded individually when lymph node metastasis is suspected based on radiographic evidence or pathological confirmed. The latest guideline for delineation of clinical target volume (CTV) for pelvic radiotherapy in patients with cervical cancer was updated by the Radiation Therapy Oncology Group (RTOG) in 2021 and highlighted that the para-aortic nodal CTV should include the lymph node groups at risk adjacent to the aorta and inferior vena cava (IVC): the paracaval, precaval, retrocaval, deep and superficial intercavo-aortic, para-aortic, preaortic, and retro-aortic nodes 15, 16. Given that irradiated areas have damage to most of tissues (from the skin through to the bone marrow) and the high sensitivity of the hematopoietic system to irradiation, bone marrow failure with neutropenia, anemia and thrombocytopenia were commonly occurred, which finally contribute to increased risk of lethal hemorrhage or infection and the interruption of chemotherapy administration or treatment failure 17, 18.

The pathogenesis of HT after irradiation is complex which remains poorly understood. Treating rats (right distal femur and proximal tibia) with irradiation at a dose of 20 Gy induced bone loss of non-irradiated bone and increased marrow adiposity at 12 weeks post-irradiation. Importantly, expression of runt-related transcription factor-2 by bone mesenchymal stem cells (BMSCs) decreased after irradiation by 88.0 % (P < 0.01) at the contralateral and 82.3 % (P < 0.01) at the irradiation site 2 weeks post-irradiation and decreased by 94.5 % (P < 0.001) at the contralateral and 44.1 % (P < 0.05) at the irradiation site 12 weeks post-irradiation, indicating that radiation-induced bone complications were partly BMSC-mediated and localized irradiation may trigger remote changes in bone which called indirect effects 19. After irradiation with a single dose of X-rays to the left hind limbs of rats, isolated BMSCs from direct and indirect irradiated bone tissue exhibited bigger cell bodies and increased granules compared to control group and the proliferation of BMMSCs decreased both in the direct irradiated and non‑irradiated bone tissue, which may be the mechanism of radiation-induced abscopal impairment to the skeleton in the cancer radiotherapy-induced bone loss 20. Increased expression of peroxisome proliferator-activated receptor gamma (PPARγ) and decreased expression of runt-related transcription factor 2 (RUNX2), accompanied by upregulated adipogenesis and downregulated osteogenesis of BMSCs as well as increased B cells and CD8+T lymphocytes in the blood were also observed at 12 weeks post-irradiation in four-month-old male Sprague-Dawley rats. These results suggested the multifaceted effects of irradiation on the blood and immune systems 21. Additionally, it has been well-discussed in a systematic review that the dose of irradiation was also identified as an influence factor of HT that needs further studied among patients with different types of cancer 22. Though it has been confirmed that prior radiotherapy does harm to bone marrow hematopoietic function of cancer patients, to date no effective drug has been reported that can help prevent bone marrow failure 23. Meanwhile, it is not yet clear whether the hematopoietic system damage induced by irradiation affect the long-term bone marrow function for cancer patients 22. Given the increasing emphasis on the quality of life of cancer patients, reducing the short-term and long-term side effects followed by radiotherapy become an urgent issue that need to be solved clinically.

To investigate and assess the effects of prior pelvic radiotherapy on long-term bone marrow function and potential risk factors of bone marrow suppression during chemotherapy in recurrent cervical cancer patients, this current study retrospectively analyzed 129 recurrent cervical cancer cases with and without prior radiotherapy, collected general clinical information, radiotherapy methods, grade bone marrow suppression of all patients and compared the rate of bone marrow suppression between the two groups by using univariable and multivariable analysis, with the hope of elucidating the risk factors of bone marrow suppression followed by chemotherapy in recurrent cervical cancer patients and trying to provide clinical evidence regarding the long-term possible effects of pelvic radiotherapy.

## Methods and materials

### Research objects

A total of 129 cases of recurrent cervical cancer that accepted primary treatment in our hospital from 2010 to 2021 were enrolled in this study, including 77 cases (59.7%) with the pelvic radiotherapy history and 52 patients (40.3%) without pelvic radiotherapy history, serving as the control group. The patients were staged according to the FIGO staging of cervical cancer in 2018 24. General information, including age, body mass index (BMI), disease free survival (DFS), FIGO stage, pathological type, treatment protocols, types of the relapse, interruption of chemotherapy due to severe bone marrow suppression, usage of bevacizumab, results of blood routine test during radiotherapy, before chemotherapy and in the period of chemotherapy after relapse were collected. Hematologic toxicity was defined by using the Common Terminology Criteria for Adverse Events (version 4.0) 25. The inclusion standards were as follows: (1) patients with cervical cancer treated in our hospital, (2) patients who chose the paclitaxel in the combination of carboplatin regimen for supplementary chemotherapy after relapse, (3) patients who formulate and finish prior radiotherapy in our hospital, (4) patients with regular follow-up. Exclusion criteria were as follows: (1) patients who didn't finish the whole treatment of supplementary chemotherapy due to various reasons, including chemotherapy allergy or serious side effects, (2) patients without regular follow-up and blood routine test in our hospital, (3) patients didn't use paclitaxel combined with carboplatin as chemotherapy regimen or change chemotherapy regimen since insensitivity, (4) patients with severe medical or surgical complications.

### Radiotherapy

**Figure 1:**
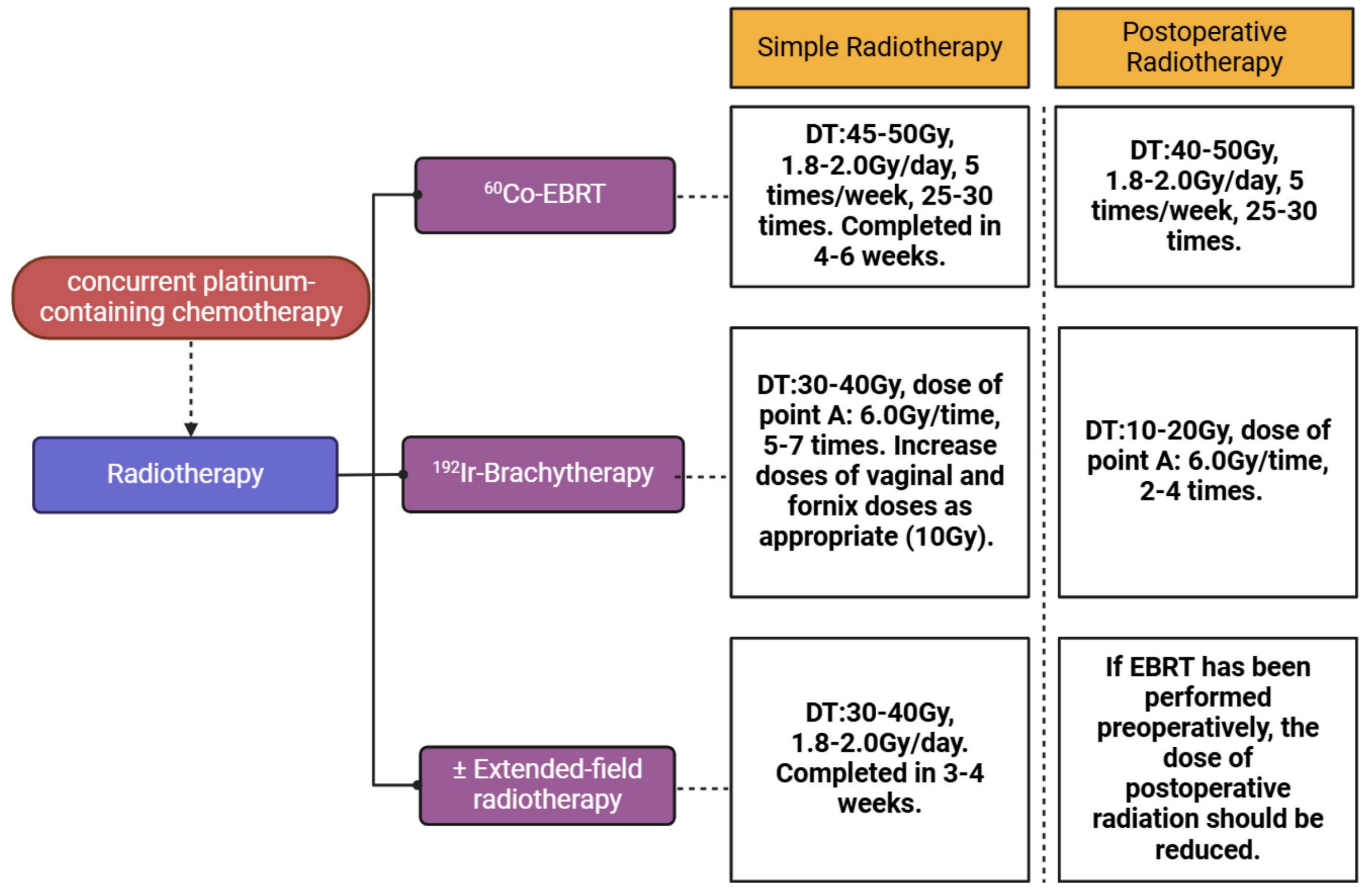
** Plan of the radiotherapy for patients with cervical cancer.** Radiotherapy for patients with cervical cancer can be divided into two groups, simple radiotherapy and postoperative radiotherapy. Each group consists of EBRT, brachytherapy and extended-field radiotherapy. The EBRT was conducted by using 60Co as radioactive source and 192Ir was used as radioactive source in brachytherapy. The dose of irradiation for EBRT and brachytherapy was based on the NCCN guideline of cervical cancer.

The prior radiotherapy plan was formulated and operated in the department of radiotherapy clinic in our hospital. Principles of radiation therapy are based on the NCCN guidelines for cervical cancer. The volume of EBRT covers the gross disease (if present), parametria, uterosacral ligaments, sufficient vaginal margin from the gross disease (at least 3 cm), presacral nodes, and other nodal volumes at risk. For patients with negative nodes on surgical or radiologic imaging, the radiation volume includes the entirety of the external iliac, internal iliac, obturator, and presacral nodal basins. For patients deemed at higher risk of lymph node involvement (eg, bulkier tumors; suspected or confirmed nodes confined to the low true pelvis), the radiation volume should be increased to cover the common iliacs as well. In patients with documented common iliac and/or para-aortic nodal involvement, extended-field pelvic and para-aortic radiotherapy is recommended, up to the level of the renal vessels (or even more cephalad as directed by involved nodal distribution). For patients with lower 1/3 vaginal involvement, the bilateral groins should be covered as well. EBRT is directed to the pelvis with or without para-aortic region and majority of patients receive concurrent platinum-containing chemotherapy during the time of EBRT. Brachytherapy is performed by using an intracavitary or an interstitial approach serving as an irreplaceable part of definitive radiotherapy. According to NCCN guidelines, in patients with an intact cervix, the dose of definitive EBRT is approximately 45 Gy (40-50 Gy) to treat primary tumor and regional lymphatics at risk and ^192^Ir-brachytherapy is used to boost primary cervical tumor with an additional 30 to 40 Gy. In highly selected, very early diseases (ie, stage IA2), brachytherapy alone (without EBRT) may be an option, especially for patients with positive vaginal margins. Extended-filed radiotherapy was applied when metastasis of para-aortic lymph node was confirmed. Patients who experienced EBRT underwent concurrent platinum-containing chemotherapy (cisplatin 40 mg per 7 days, 4-6 times) to increase the sensitivity of radiotherapy. The detailed plan of radiotherapy was presented in Figure 1.

Determination of radiation field: The patient drank water one hour before positioning CT scan until the bladder was filled and was placed in a supine position with both hands holding their heads. The position was fixed with a vacuum pad and a thermoplastic mold, and enhanced CT scan was used for positioning with a slice spacing of 5mm. After scanning, transfer the image data to the radiotherapy planning system for target area delineation 26, 27.

Definition of target area for curative radiotherapy: Gross tumor volume (GTV) is the primary tumor based on enhanced MRI/CT or PET-CT imaging; GTVnd: Metastatic lymph nodes in the pelvic cavity or adjacent to the abdominal aorta as indicated by imaging examination; CTV1: including GTV + uterine body + cervix; CTV2: including parametrial/vaginal tissue, bilateral adnexa, and proximal vagina (if the vagina is not invaded, including 1/2 of the upper segment; if the upper segment of the vagina is invaded, including 2/3 of the upper segment; if the vagina is extensively invaded, including the entire vagina); CTV3: including the iliac, iliac, iliac, sacral, and obturator lymph drainage areas and patients with clear or high-risk lymph node metastasis in the paraaortic region should include the paraaortic lymph drainage area 26, 27.

### Chemotherapy

For chemotherapy, if surgical patients have high-risk factors (such as parametrial invasion, positive lymph nodes, positive surgical margins, and adverse pathological types) after primary surgery, sequential radiotherapy and chemotherapy (4-6 times of platinum containing chemotherapy) will be supplemented. When recurrence was confirmed, all of the patients underwent a chemotherapy regimen of paclitaxel combined with carboplatin, with a carboplatin dose of area under the curve (AUC) 5 to AUC6 and a paclitaxel dose of (135-175) mg / m^2^ * body surface area (m^2^) per 21 days for 5 to 6 times totally. Patients who underwent combination treatment with bevacizumab received simultaneously administering bevacizumab on the day of chemotherapy. The dose of bevacizumab was 7.5mg/kg*body weight (kg). The decision on the dosage of chemotherapy drugs and bevacizumab was made by two attending doctors with at least 5 years of experience in the treatment of gynecological oncology in our hospital.

### Blood parameter analysis

Hematological toxicity refers to the decrease in blood cell count caused by radiation induced decrease in bone marrow hematopoietic function. According to the World Health Organization's classification criteria for acute and subacute hematological toxic reactions to the treatment, we assessed the bone marrow function of patients by doing routine blood test weekly. Given that hematological toxicities decrease production of red blood cells (anemia), production of white blood cells (neutropenia or granulocytopenia), and production of platelets (thrombocytopenia), we collected the information of hemoglobin (HGB), White Cell Count (WCC), Absolute Neutral Count (ANC), and Platelets (PLT) of each patient and the lowest blood cell count for each patient during radiotherapy was recorded and used to analyze further (Table 1) 11, 28.

HGB, WCC, ANC and PLT were determined from blood samples collected at baseline and weekly before and in the period of entire chemotherapy. Maximum toxicity grading and the type of HT during previous radiotherapy and chemotherapy at relapse were noted for each patient.

### Follow-up checkups

The follow-up was scheduled for 3 to 6 months within the first three years after surgery or radical radiotherapy and then every 6 months for the next two years, and one for every year after 5 years. Physical examination, gynecologic examination, thinprep cytologic test (TCT), imaging examination (X-ray and pelvic ultrasound), computed tomography (CT) or magnetic resonance imaging (MRI) are selected to assess disease condition for every follow-up. All cases were under regular follow-up. Disease free survival of patients was also collected, which refers to the time from disease-free survival after surgery to the occurrence of disease recurrence or metastasis.

### Statistical Analysis

SPSS 26.0 was used for data analysis. The measurement data of normal distribution were expressed as the mean ± standard deviation. Descriptive statistics were performed on all variables. Comparisons between the two groups were performed with the *t* test, analysis of variance (ANOVA) and *χ^2^*-test, as appropriate. A logistic regression model was used for multivariable analysis. GraphPad Prism 8 (La Jolla, CA, USA) was employed to present all graphs. P values of < 0.05 were considered to have significant group differences.

## Results

### General clinical information of recurrent cervical cancer patients

A total of 129 patients with recurrent cervical cancer were included in this study. All of these patients were primarily diagnosed and treated in our hospital with 77 cases had radiotherapy history at the point of recurrence, whereas 52 cases didn't undergo prior radiotherapy were used as control group. The age, BMI, DFS, FIGO stages, pathological types, radiotherapy methods, bone marrow suppression during previous radiotherapy, extended-field radiotherapy, relapse site, completion of chemotherapy after relapse, usage of bevacizumab was collected. The general clinical information of the two groups is listed in Table 2. No significant differences were observed between the two groups in age, BMI, DFS and pathological types (P > 0.05). FIGO stages and radiotherapy methods were significantly different between the two groups (P < 0.05). The proportion of patients with stages I-III in the radiation group was far higher than that in no radiotherapy history group. By utilizing blood routine test, 50.5% (n=39) of patients with previous radiotherapy exhibited bone marrow suppression in the period of radiotherapy, whereas 49.5% didn't showed hematological events (P>0.05). For patients with history of previous radiotherapy, 6.5% (n=5) of them underwent extended field irradiation due to para-aorta lymph node metastasis and 71.4% (n=55) of them were found to relapse inside the previous irradiated field, whereas 28.6% (n=22) of them relapsed outside the previous irradiated field. Notably, patients who experienced severe bone marrow suppression may face to anemia, infection or even lethal bleeding which force them to interrupt chemotherapy. Our results showed that 16.9% and 5.8% of patients with and without previous radiotherapy interrupted chemotherapy due to severe bone marrow suppression (P=0.060). Besides, there were 13.0% and 13.5% of patients underwent chemotherapy combined with bevacizumab after recurrence in the radiation group and control group, respectively.

### Comparison of HT3+ risk between two groups by univariable analysis

According to the grading criteria for bone marrow suppression, we defined grade 3 and grade 4 bone marrow suppression as HT3+, which were considered severe side effects of chemotherapy, and analyzed the incidence of HT3+ in patients with or without previous radiotherapy, The result showed that patients with prior radiotherapy history exhibited higher risk of HT3+ during chemotherapy, compared with those without prior radiotherapy history (*χ^2^*=12.939, P = 0.005). Furthermore, patients accepted brachytherapy combined with EBRT showed the highest incidence of HT3+ (44.9%), compared to that in patients with brachytherapy alone (25.0%) and EBRT alone (40.0%). Given that equal or over grade 3 HT (HT3+) required clinical intervention and treatment, we analyzed the potential risk factors for HT3+ in recurrent cervical cancer patients with or without radiotherapy history by using *t* test, ANOVA, and *χ^2^*-test, as appropriate. The age, BMI, DFS, FIGO stages, pathological types and methods of radiotherapy, bone marrow suppression during previous radiotherapy were included (Table 2). Univariable analysis results showed that age, BMI, DFS, FIGO stages and pathological types had no significant differences between patients with or without HT3+ during the period of chemotherapy after recurrence (P > 0.05). For 39 recurrent patients have bone marrow suppression during the previous radiotherapy, 18 cases (18/39, 46.2%) of them exhibited HT3+ in the period of chemotherapy after relapse, whereas 15 cases (15/38, 39.5%) of no-bone marrow suppression during the previous radiotherapy exhibited HT3+ in the period of chemotherapy after relapse (*χ^2^*=0.351, P = 0.554). Combination of bevacizumab with chemotherapy didn't increase the incidence of HT3+ in recurrent cancer patients (P=0.626). When there is suspicion or postoperative confirmation of paraaortic lymph node metastasis, extended-field radiotherapy is required to achieve satisfactory therapeutic effects. In our study, there were 5 patients who underwent extended-field radiotherapy and 80.0% (n=4) of these 5 patients experienced HT3+ in the period of chemotherapy, whereas 40.3% (29/43) of patients without extended-field radiotherapy exhibited HT3+ (P=0.083). Although the difference between the two groups was not statistically significant, patients with extended-field radiotherapy have a higher trend of severe bone marrow suppression. The possible explanation of this phenomenon was that extended-field radiotherapy leads to further accumulation of doses of bone marrow and a decrease in bone marrow function, suggesting that patients with a history of extended-field radiotherapy should be more vigilant about the risk of bone marrow suppression.

To assess whether the baseline of WCC, HGB and PLT affecting the incidence of HT3+, we calculated the accounts of WCC, HGB and PLT before the first treatment of chemotherapy at relapse by using blood routine test (Table 3). Beyond expectation, the baseline of WCC, HGB and PLT before chemotherapy at relapse in patients with or without HT3+ showed no significant differences, indicating that the baseline of blood cells was not related to the risk of HT3+ for cervical cancer patients.

### Comparison of HT3+ risk between two groups by multivariable analysis

To further evaluate the relationship between the above factors and the risk of HT3+ in recurrent cervical cancer patients during chemotherapy, binary logistic regression model was employed to perform multivariable analysis. The final logistic model is statistically significant, *χ^2^*=16.975, P=0.001). Similar to the results of univariable analysis, prior radiotherapy and extended-field radiotherapy were independent risk factor of HT3+, and the OR of brachytherapy, EBRT and brachytherapy combined with EBRT were 3.884, 9.410 and 15.462, respectively. Extended-field radiotherapy also increased the risk of HT3+ and the OR of it was 15.320. The model can correctly classify 69.8% of the research objects. The other factors in the model did not show significant impact on severe bone marrow suppression for recurrent cervical cancer patients (Table 4).

### The relationship between baseline of blood cell levels post-radiation and blood events during chemotherapy

To investigate whether the history of pelvic radiotherapy affect the baseline of blood cells, including WCC, HGB and PLT, we collected the relative information by using blood routine test of cervical cancer patients with or without history of radiotherapy before the first course of chemotherapy after relapse. The baseline of three types of blood cells was shown in the Table 5 as mean ± SD in two groups. Results showed that there were no differences between the baseline of patients with or without history of pelvic radiotherapy in the counts of WCC, HGB and PLT.

**Figure 2:**
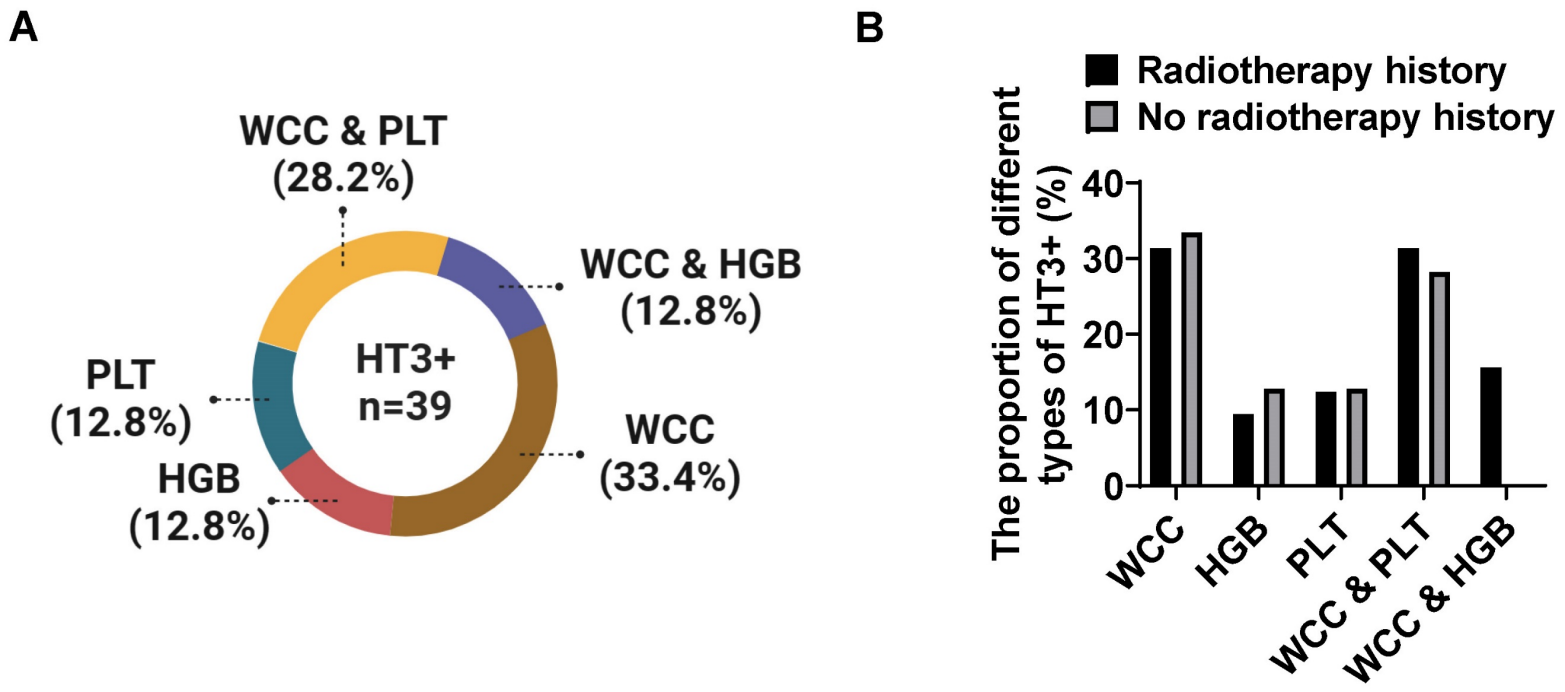
** The proportion of different types of severe hematological events in recurrent cervical cancer patients.** There were 39 patients experienced grade III and IV bone marrow suppression during chemotherapy in this study and 33.4% of patients showed decreased WCC, 12.8% of patients exhibited reduced HGB and 12.8% of patients were detected decreased PLT alone in blood routine tests, whereas 40.0% patients showed severe reduction in more than one type of blood cell (A). Prior radiotherapy showed no significant effects on the types of HT3+ in patients with grade III and IV bone marrow suppression (B).

We also analyzed the type of HT3+ in recurrent cervical cancer patients with HT3+. A total of 39 cases experienced HT3+ during chemotherapy after relapse, of which 32 cases (82.1%) had prior radiotherapy history and 7 cases without. Among these 39 cases, 33.4% (n=13) of patients showed severe decreased WCC during chemotherapy, 12.8% (n=5) of patients showed decreased HGB alone, 12.8% (n=5) of patients exhibited reduced PLT alone, 28.2% (n=11) of patients showed decreased WCC combined with PLT, and the rest (12.8%, n=5) showed decreased WCC combined with HGB (Figure 2A). The proportion of different types of HT3+ was similar between the two groups (Figure 2B). Although both WCC and PLT showed a high probability of reduction, our data showed a significant decrease in WCC, and the trend of decreasing blood cell types tends to be the same between the two groups.

## Discussion

Radiation therapy is the main treatment for cervical cancer patients at early stages with high risk factors confirmed by postoperative pathology and for those at late stages (IIB-IV). The bone marrow, as a hematopoietic organ, consists of up to 50% hematopoietic site in adults and is highly sensitive to radiotherapy. Exposure of bone marrow microvasculature to irradiation not only hinders the blood supply to the bone marrow, but also has direct or indirect adverse effects on other normal physiological functions 29. In an animal model, the dynamic balance between the internal and external blood vessels in the bone marrow of mice is disrupted after one week of radiation with 9.5Gy. Further study suggested that ionizing radiation damaged the sinusoids of the bone marrow and caused dilation and bleeding of the sinusoids 30. In addition to the reduction of lymphocytes caused by short-term ischemia or direct radiation damage, the long-term changes in bone tissue after radiotherapy are due to a decrease in the number of bone marrow cells at the radiation therapy site, which are replaced by adipose tissue, resulting in new and progressive bone marrow replacement, tumor recurrence, or radiation induced osteonecrosis after radiation therapy 31. Patients with pelvic lymph node metastasis of cervical cancer have a larger target area and higher dose compared to those without lymph node metastasis and these patients are more susceptible to extensive and high-dose irradiation of the bone marrow, and combined with synchronous platinum chemotherapy, acute bone marrow suppression can occur in a short period of time, thereby affecting the treatment effectiveness of patients 27, 32. In this study, we analyzed the effects of prior radiotherapy on the risk of bone marrow suppression in recurrent cervical cancer cases and found that the history of pelvic radiotherapy significantly increased the incidence of bone marrow suppression during chemotherapy in recurrent patients. A total of 77 recurrent patients have received prior radiotherapy in this study, of which 32 (41.6%) had severe bone marrow suppression (grade III-IV) during TC regimen chemotherapy after recurrence, whereas 13.3% of patients (4/52) with no prior radiotherapy exhibited grade III-IV bone marrow suppression. Our results showed that both univariable and multivariable analysis showed that the history of prior radiotherapy was an independent risk factor for HT3+ in recurrent cervical cancer patients undergoing TC regimen chemotherapy (P < 0.05). Meanwhile, extended-field radiotherapy also increases the risk of severe bone marrow suppression in recurrent patients to a certain extent (80.0% vs 40.3%, P < 0.05). Additionally, based on our results, combination of bevacizumab and chemotherapy did not increase the risk of HT3+ of recurrent patients with cervical cancer by using univariable and multivariable analysis. These results indicated that prior pelvic radiotherapy had a long-term effect on the function of pelvic bone marrow and patients with prior radiotherapy were more prone to severe bone marrow suppression. Although, in the study, we were unable to measure the percentage increase in dose received by the active bone marrow from e extended-field radiotherapy compared to normal area of pelvic irradiation, our results pointed out that patients with cervical cancer who received extended-field radiotherapy had higher risk for bone marrow suppression when facing the challenge of hematological toxicities.

The ability of bone marrow to regenerate after pelvic radiation depends on the amount of radiation it receives 33. Notably, when high-dose radiotherapy is used, the bone marrow requires more time to repair, and some bone marrow injuries are irreversible 34. Increased irradiation dose of pelvic was associated with reduced nadir white cell count and absolute neutrophil count in patients with anal cancer and led to a higher risk of early hematological adverse events grade 3 or greater 33. A retrospective study evaluated the hematological events of 35 patients with locally advanced rectal cancer who received preoperative radiotherapy followed by postoperative 5-fluorouracil combined oxaliplatin (oxf) chemotherapy during treatment. The results showed that preoperative radiotherapy had a lasting effect on the function of pelvic bone marrow and greatly increased the risk of grade ≥ 3 hematological toxicities during postoperative chemotherapy 35. In patients with locally advanced cervical cancer who received concurrent chemoradiotherapy, three different irradiation doses for fractionated treatment, 10 Gy, 20 Gy and 40 Gy, were shown to be related to the risk of hematological toxicity and 20 Gy was identified as the most significant toxic radiotherapy dosage threshold clinically 22. Compared to cervical cancer patients with pelvic bone V10 < 90%, patients with pelvic bone V10 ≥ 90% had a higher proportion of grade 2 or higher leukopenia and neutropenia 36. In esophageal cancer patients receiving radiotherapy and chemotherapy, the relative volume size of sternum bone marrow irradiated with over 20 Gy dose of radiation was significantly negatively correlated with the level of peripheral blood lymphocytes 37. Conducting various bone structure dosimetry parameters related to acute hematological toxicity in patients with anal squamous cell carcinoma receiving radiotherapy and chemotherapy, the results showed a positive relationship between average dose in the entire bone or bone marrow cavity of the lumbosacral vertebrae and the risk of grade 3 and above hematological toxicity above level 3 38. In our study, patients receiving brachytherapy with lower radiation doses had a lower risk of bone marrow suppression during subsequent chemotherapy compared to those received EBRT (25.0% vs 40.0%, P<0.05). Our findings emphasized the long-term damage that traditional two-dimensional pelvic radiation caused to the pelvic bone marrow of cancer patients, and due to the need for treatment, the dosage used in radiotherapy cannot be reduced. The improvement of radiotherapy technology may be an effective way to solve this problem.

With the progress of radiotherapy technology, intensity modulated radiotherapy (IMRT) and three-dimensional conformal radiotherapy, which can individualize irradiation area and dose, are gradually applied to clinics and are identified with lower risk of HT3+. For 231 patients with anal cancer who received radiotherapy, IMRT effectively reduced the hematologic toxicity caused by radiotherapy and minimize the interruption of treatment caused by acute toxicity compared with traditional technology 39. Importantly, IMRT was better than 3D-conformal technology in reducing the acute radiotherapy response of large intestine and small intestine in patients with cervical cancer and endometrial cancer 31. A single center study also found that the application of pelvic bone marrow sparing IMRT can reduce hematologic toxicity. The incidence of equal or over grade II hematologic toxicity (50.0%) was significantly lower than that of the control group (69.5%), P=0.02 40. Despite IMRT significantly reduced radiotherapy dose of pelvic bone marrow, its ability to reducing hematological toxicity after fluorothymidine-18 based chemotherapy remained challenging for the slow inhibition of bone marrow activity with an irradiation dose greater than 35 Gy in patients with pelvic cancer (n=32) 41. These results emphasized the benefits of new radiotherapy technologies, including IMRT in reducing bone marrow damage caused by radiotherapy. Although the current evidence on how much dose received by active bone marrow can be reduced by IMRT and its association with radiotherapy regimens is still limited, this is positive news for reducing the short- and long-term pelvic bone marrow suppression in cervical cancer patients.

Neutropenia or deficiency is a common complication of chemotherapy. According to existing retrospective studies, chemotherapy-induced neutropenia occurred in nearly 50.5% (n=147) gynecologic patients over 23.4% (378) chemotherapy cycles, associating with older age (over 70 years), less than five previous chemotherapy cycles, disseminated disease, platinum-based regimens and taxane-containing regimens 42. In this study, all recurrent cervical cancer patients have received platinum-based and paclitaxel-containing combined chemotherapy after recurrence and 17 patients underwent a combination treatment of bevacizumab in addition to chemotherapy. Compared to patients without prior pelvic radiotherapy, prior pelvic radiotherapy did not reduce the baseline of blood cell (WBC, nadir ALC, HGB and PLT) significantly (P > 0.05) of cancer patients. In 39 cases with HT3+ during chemotherapy after relapse, 33.4% (n=13), 12.8% (n=5), 12.8% (n=5) patients experienced sharp reduction in WCC, HGB and PLT, respectively. Besides, another 28.2% (n=11) and12.8% (n=5) of HT3+ cases exhibited combined reduction in WCC and PLT, WCC and HGB, respectively. Given the severe hematologic toxicity, 12.4% of patients (16/129) experienced unexpected interruption of chemotherapy due to severe bone marrow suppression. Among them, severely decreased WCC and PLT were the most common factors that limiting chemotherapy when monitored hematopoietic capacity of bone marrow by using weekly blood routine test in the period of chemotherapy. Severe leukopenia and thrombocytopenia can cause fever, infection, and fatal bleeding, which not only increases the readmission rate of patients, but also increases the medical and economic burden. Optimistically, the usage of hematopoietic colony-stimulating factors (CSFs) in clinical has gradually become universal, and their application in cancer patients has greatly reduced the incidence of hematological complications after chemotherapy 43. Therefore, more attention should be paid to hematological complications in patients with a history of pelvic radiation therapy and promptly consider the use of CSFs.

## Conclusion

In conclusion, our current findings demonstrate, for the first time, that pelvic radiotherapy increased the risk of severe HT and lead to interruptions of chemotherapy for recurrent cervical cancer patients. Extended-field radiotherapy was also a risk factor for HT3+, while combination of bevacizumab and platinum-based chemotherapy showed no significant impacts on the incidence of HT3+. Moreover, WCC and PLT were the main blood indicators affected during chemotherapy after relapse. Little effects of prior pelvic radiotherapy for patients with cervical cancer on the long-term baseline counts of blood cells were observed. Our results identified the risk factors of sever HT of recurrent cervical cancer patients and provided a limited long-term observation of the impact of pelvic radiotherapy on bone marrow function, with the hope of promoting the understanding of treatment for recurrent cervical cancer patients.

## Figures and Tables

**Table 1:** Criteria for acute and subacute hematological toxicity to the treatment

Hematological toxicity	Normal	Grade I	Grade II	Grade III	Grade IV
HGB (g/L)	≥110	<110 and ≥95	<95 and ≥80	<80 and ≥65	<65
WCC (*10^9^/L)	≥4.0	<4.0 and ≥3.0	<3.0 and ≥2.0	<2.0 and ≥1.0	<1.0
ANC (*10^9^/L)	≥2.0	<2.0 and ≥1.5	<1.5 and ≥1.0	<1.0 and ≥0.5	<0.5
PLT (*10^9^/L)	≥100	<100 and ≥75	<75 and ≥50	<50 and ≥25	<25

**Table 2:** Comparison of general information between the two groups

Characteristics	Patients with radiotherapy history (n=77) (%)	No radiotherapy history (n=52) (%)	P value
**Age (years)**	50.3±11.5	47.5±8.9	0.139
**BMI (Kg/m^2^)**	23.9±3.8	24.2±3.2	0.605
**DFS (months)**	26.7±27.7	52.2±22.9	0.000
**FIGO stages**			0.000
I	22 (28.6)	41 (78.8)	
II	36 (46.8)	10 (19.2)	
III	17 (22.1)	1 (1.9)	
IV	2 (2.6)	0 (0)	
**Pathlogical types**			0.938
Squamous carcinoma	67 (87.0)	27 (86.5)	
Adenocarcinoma	10 (13.0)	7 (13.5)	
**Radiotherapy methods**			
Brachytherapy	8 (10.4)	0	
EBRT	20 (26.0)	0	
Brachytherapy + EBRT	49 (63.6)	0	
**Bone marrow suppression** **during previous radiotherapy**			
Yes	39 (50.6)	0	
No	38 (49.4)	0	
**Extended-field radiotherapy**			
Yes	5 (6.5)	0	
No	72 (93.5)	0	
**Relapse site inside the** **previous irradiated field**			
Yes	55 (71.4)	0	
No	22 (28.6)	0	
**Interruption of chemotherapy** **due to severe BMS**			0.060
Yes	13 (16.9)	3 (5.8)	
No	64 (83.1)	49 (94.2)	
**Usage of bevacizumab**			0.938
Yes	10 (13.0)	7 (13.5)	
No	67 (87.0)	42 (86.5)	

**Table 3:** Univariable analysis of risk factors and HT3+

	HT 3+		*t*/Z or *χ^2^*	P value
	Positive (%)	Negative (%)		
**Age (years)**			0.404	0.525
<60	26 (28.6)	65 (71.4)		
≥60	13 (34.2)	25 (65.8)		
**BMI (Kg/m^2^)**			0.111	0.739
<30	37 (30.6)	84 (69.4)		
≥30	2 (25.0)	6 (75.0)		
**DFS (months)**	34.7±33.7	38.0±26.3		0.552
**FIGO stages**			0.864	0.834
I	17 (27.0)	46 (73.0)		
II	15 (32.6)	31 (67.4)		
III	6 (33.3)	12 (66.7)		
IV	1 (50)	1 (50)		
**Pathlogical types**			1.112	0.292
Squamous carcinoma	32 (28.6)	80 (71.4)		
Adenocarcinoma	7 (41.2)	10 (58.8)		
**Radiotherapy methods**			12.939	0.005
No prior radiotherapy	7 (13.5)	45 (86.5)		
Pelvic radiotherapy history	32 (41.6)	45 (58.4)		
Brachytherapy	2 (25.0)	6 (75.0)		
EBRT	8 (40.0)	12 (60.0)		
Brachytherapy + EBRT	22 (44.9)	27 (55.1)		
**Bone marrow suppression during previous radiotherapy** **(n=77)**			0.351	0.554
Yes	18 (46.2)	21 (53.8)		
No	15 (39.5)	23 (60.5)		
**Usage of bevacizumab**			0.283	0.626
Yes	6 (35.3)	11 (64.7)		
No	33 (29.5)	79 (70.5)		
**Extended-field radiotherapy** **(n=77)**			3.012	0.083
Yes	4 (80.0)	1 (20.0)		
No	29 (40.3)	43 (59.7)		
**Counts of WCC before chemotherapy at relapse** **(Mean ± SD) (10^9^/L)**	6.38±2.44	6.48±1.92	0.240	0.811
**Counts of HGB before chemotherapy at relapse** **(Mean ± SD) (g/L)**	124.21±14.21	126.42±11.53	0.933	0.352
**Counts of PLT before chemotherapy at relapse** **(Mean ± SD) (10^9^/L)**	278.82±94.06	268.44±77.38	-0.654	0.514

**Table 4:** Multivariable analysis of HT3+ and radiotherapy methods

	β	SE	Wald	df	OR (95%CI)	P value
**Age (years)**	0.006	0.022	0.083	1	1.006 (0.964-1.050)	0.774
**BMI (kg/m^2^)**	-0.055	0.068	0.647	1	0.947 (0.829-1.082)	0.421
**DFS (months)**	0.008	0.009	0.859	1	1.008 (0.991-1.025)	0.354
**FIGO stages (I)**			3.583	3		0.310
II	-0.980	0.630	2.422	1	0.375 (0.109-1.289)	0.120
III	-1.203	0.899	1.791	1	0.300 (0.052-1.748)	0.181
IV	-3.013	2.188	1.897	1	0.049 (0.001-3.578)	0.168
**Pathological types** **(Adenocarcinoma)**	-0.945	0.666	2.012	1	0.389 (0.105-1.434)	0.156
**Radiotherapy methods**			11.470	3		0.009
Brachytherapy	1.357	1.175	1.333	1	3.884(0.388-38.883)	0.248
EBRT	2.242	0.797	7.907	1	9.410(1.972-44.983)	0.005
Brachytherapy + EBRT	2.738	0.856	10.232	1	15.462(2.888-82.789)	0.001
**Extended-field radiotherapy**	2.729	1.307	4.362	1	15.320(1.183-198.438)	0.037
**Bone marrow suppression during previous radiotherapy**	0.003	0.566	0.000	1	1.003(0.331-3.040)	0.995
**Usage of bevacizumab**	0.672	0.688	0.955	1	1.959(0.509-7.539)	0.328
**Counts of WCC before chemotherapy at relapse (10^9^/L)**	0.023	0.112	0.041	1	1.023(0.821-1.275)	0.840
**Counts of HGB before chemotherapy at relapse (g/L)**	-0.012	0.020	0.347	1	0.988(0.949-1.028)	0.556
**Counts of PLT before chemotherapy at relapse (10^9^/L)**	0.002	0.003	0.385	1	1.002(0.996-1.008)	0.535
**Constant**	-0.719	2.417	0.044	1	0.487	0.833

**Table 5:** The absolute values of WCC, HGB and PLT in patients with or without radiotherapy at relapse

	Patients with history of radiotherapy (n=77)	Patients without history of radiotherapy (n=52)	t	P value
**WCC (10^9^/L)**	6.42 ± 2.27	6.49 ± 1.78	0.316	0.753
**HGB (g/L)**	126.0 ± 13.94	125.4 ± 9.86	-0.305	0.761
**PLT (10^9^/L)**	270.1 ± 83.2	273.7 ± 82.3	0.361	0.719

## Abbreviations

HPV: human sustainable papillomavirus
EBRT: external beam radiation therapy
Gy: gary
NCCN: national comprehensive cancer network; HT: hematologic toxicity
CTV: clinical target volume
RTOG: radiation therapy oncology group
IVC: inferior vena cava
BMSC: bone mesenchymal stem cell
PPARγ: peroxisome proliferator-activated receptor gamma
RUNX2: runt-related transcription factor 2
DFS: disease free survival
BMI: body mass index
FIGO: federation international of gynecology and obstetrics
GTV: gross tumor volume
AUC: area under the curve
HGB: hemoglobin
WCC: white cell count
ANC: absolute neutral count
PLT: platelet
TCT: thinprep cytologic test
CT: computed tomography
Oxf: oxaliplatin
IMRT: intensity modulated radiotherapy
CSFs: colony-stimulating factors
